# *In Vitro* Antiviral Activity of some Novel Isatin Derivatives against HCV and SARS-CoV Viruses

**DOI:** 10.4103/0250-474X.40339

**Published:** 2008

**Authors:** P. Selvam, N. Murgesh, M. Chandramohan, E. De Clercq, E. Keyaerts, L. Vijgen, P. Maes, J. Neyts, M. V. Ranst

**Affiliations:** Arulmigu Kalasalingam College of Pharmacy, Krishnankoil - 626 190, India; 1 Institute of Pharmacology, Madurai Medical College, Madurai - 625 020, India; 2 Bharat Ratna Kamarajar Liver Hospital and Research Center, Madurai - 625 001, Belgium; 3 Raga Institute for Medical Research, Katholieke Universiteit-Leuven, Minder broederstraat 10, LeuvenB-3000, Belgium

**Keywords:** Isatin, HCV, SARS-CoV, vero cells, huh 5-2 cells

## Abstract

4-[(1,2-dihydro-2-oxo-3H-indol-3-ylidene)amino]-N(4,6-dimethyl-2-pyrimidiny)benzene sulphonamide and its derivatives were evaluated for antiviral activity against Pathogenic viruses such as Hepatitis C Virus and SARS-CoV in Vero and Huh 5-2 cells, respectively. The 5-fluoro derivative inhibited the HCV RNA synthesis at 6 μg/ml, without toxicity at a concentration up to 42 μg/ml in Huh 5-2 cells. Among the compounds tested SPIII-5F exhibits the 45% maximum protection against replication of SARS-CoV in Vero cells.

Isatin (2,3-dioxoindole), a versatile lead molecule for potential bioactive agents, and its derivatives were reported to posses anticancer^1^, antibacterial activities^2^–^4^. Methisazone (N-methylisatin-β-thiosemicarbazone) was one of the first clinically used synthetic antiviral agent^5^. Isatin derivative were reported for antiviral activity against a verity of pathogens viruses^6^ and N,N-disubstitutedthiosemicarbazone derivative of isatin were tested for inhibition of HIV-1 replication^7^. Previously we reported synthesis of novel isatin derivatives and evaluated antiviral activity against HIV-1 and HIV-2 in MT-4 cells^8^. Significant antiviral activity was observed with these compounds against HIV-1 replication^9^.

In view of the broad spectrum activities of isatin derivatives, we aimed at evaluating the antiviral activity of some novel 4-1[(1,2-dihydro-2-oxo-3H-indol-3-ylidene)amino]-N(4,6-dimethyl-2-pyrimidiny)-benzenesulphonamide and its derivatives (fig. 1) against pathogenic viruses such as hepatitis C virus (HCV) in human hepatoblastoma cells (Huh 5-2 cells) and Severe Acute Respiratory Syndrome corona virus (SARS-CoV) in Vero cultures.

**Fig. 1:**
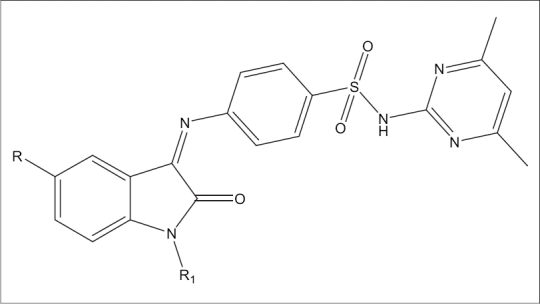
N-(4,6-dimethylpyridin-2-yl)-4-(2-oxo-1,2-dihydroindol-3- ylideneamino) benzenesulfonamide and its derivatives. R and R_1_ for SPIII-5Br are Br and H, for SPIII-5Cl are Cl and H, for SPIII-5F are F and H, for SPIII-5H are H and H, for SPIII-Me are CH_3_ and H and for SPIII-NA are H and COCH_3_, respectively.

4-[(1,2-dihydro-2-oxo-3H-indol-3-ylidene)amino]-N(4,6-dimethyl-2-pyrimidiny)benzene sulphonamide and its derivatives (fig. 1) were prepared by condensing the isatin and its derivatives (5-chloro, 5-bromo, 5-flouro, 5-methyl and N-acetyl) with sulphadimidine in the presence of glacial acetic acid^9^.

In the method adopted for antiviral activity against SARS-CoV in vero cells^10^. Vero E6 cells in 96-well tissue culture plates were used confluent. Culture medium was removed and 100 μL of minimum essential medium supplemented with 2% fetal bovine serum containing an appropriate concentration of antiviral compound was added. Inside a biosafety laboratory-3,25 μL of a SARS-CoV virus solution added. Five concentrations were tested for cytotoxicity of the antiviral compounds. After an incubation period of three days at 37° in 5% CO_2_, the inhibition of the cytopathic effect (CPE) by the compounds was measured in a spectrophotometer (at 492) by the reduction by cellular dehydrogenase of the 3-(4,5-dimethylthiazol-2-yl)-5-(3-carboxymethoxy-phenyl)-2-(4-sulfophenyl)-2H-tetrzolium(MTS) dye (Cell titer 96 Aqueous One Solution kit, promega) (20 μL MTS for 3h at 37°) in to a water soluble coloured formazan product. The antiviral activity and cytotoxcity of the test compounds are presented in Table 1.

**TABLE 1:** ANTIVIRAL ACTIVITY OF ISATIN DERIVATIVE AGAINST SARS-COV IN VERO E6 CELLS

Compound code	EC_50_^a^ (μg/ml)	CC_50_^b^ (μg/ml)	Maximum protection (%) (at 125 μg/ml)
5CI-IS-AC	>125	>125	0
SPIII-5H	>125	>125	22
SPIII-5Cl	>125	>125	10
SPIII-5Br	>125	>125	2
SPIII-5F	>125	>125	45
SPIII-5Me	>125	>125	12
SPIII-NA	>125	>125	12

a 50% effective concentration required to reduce virus-induced cytopathicity by 50%.

b 50% cytotoxic concentration required to reduce host cell viability by 50%

Replication assay undertaken with Huh-5-2 cells^11^–^13^ [a cell line with a persistent HCV replication 1389luc-ubi-neo/NS3-3/5.1; replication with firefly luciferase-lubquitin-neomycine phosphotransferase fusion protein EMCV-IRES drivan NS3-5B HCV polyprotein] was cultured in RPMI medium 2 mM glutamine, 1× non essential amino acid (Life Technologies, DC); 100 IU/ml penicillin and 100 μg/ml streptomycin and 250 μg/ml G418 (Geneticin, Life Technologies Washington DC). Cells were seeded at a density of 7000 cells per well in 96 well view plate TM (Packard, CA) in medium containing the same compounds as described above, except for G418. Cells were allowed to adhere and proliferate for 24 h. At that time, culture was removed and serial dilution of test compounds were added in culture medium lacking G418. Interferon alfa 2a (500 IU) was added as a positive control. Plates were further incubated at 37° and 5% CO_2_ for 72 h replication of HCV replicon in Huh-5 cells results in luciferase activity in the cells. Luciferase activity was measured by adding 50 μl of 1 × Gloysis buffer (Promega) for 15 min of followed by adding 50 μl Steady-Glo Luciferase assay reagent (promega). Luciferase activity was measured with luminometer and signal in each individual well was expressed as a percentage of the untreated culture. Parallel culture of Huh 5-2 cells, seeded at a density of 7000 cells/well of classical 96-well cell culture plates (Becton-Dicknson) were treated in a similar fashion except that no Glo-lysis buffer or Stady-Glo Luciferase reagent was added. Instead the density of the cluture was measured by means of the MTS method (Promega). The antiviral activity and cytotoxicity of the test compounds are prepared in Table 2.

**TABLE 2:** ANTI-HCV ACTIVITY OF ISATIN DERIVATIVES

Compound code	EC_50_ (μg/ml) HCV RNA*	CC_50_ (μg/ml) Cell growth*	SI
5CI-IS-AC	>50	50	0
SPIII-5H	19	>50	>2
SPIII-5CI	>50	>50	0
SPIII-5BR	17	>50	>3
SPIII-5F	6	42	7
SPIII-5Me	>50	>50	0
SPIII-NA	>50	>50	0

* % untreted control. Interferon alfa-2b at 10.000 units/well reduced the signal in the viral RNA (luciferase) assay to background levels; without any cytostatic activity

From the antiviral and cytotoxicity assay it was observed that the compounds 4-[(1,2-dihydro-2-oxo-3H-indol-3-ylidene)amino]-N(4,6-dimethyl-2-pyrimidiny)benzene sulphonamide (SPIII-5H) and bromo derivative (SPIII-Br) inhibits HCV RNA synthesis at the EC_50_ of 17 and 19 μg/ml, respectively while its CC_50_ for cell growth was 42 μg/ml in Huh 5-2 cells. The isatin lead molecule 5Cl-IS-AC did not inhibit the HCV RNA synthesis (EC_50_ and CC_50_ more than 50 μg/ml) and the replication of SARS-CoV (maximum protection 0%). SPIII derivative showed 2-45% maximum protection against the replication of SARS-CoV in Vero cells and compound SPIII 5F exhibited 45% maximum protection against the replication of acutely infected SARS-CoV in Vero cell.

The present study was aimed at investigating some novel isatin derivative for antiviral activities against HCV and SARS-CoV to identify potential bioactive agent in the series. From the results of biological activities it appeared that some of the derivatives showed antiviral activity against HCV virus in Huh 5-3 cells.SPIII-5H and bromo derivatives inhibited the synthesis of HCV RNA, but only at a relatively high concentration (17 and 19 μl/ml).

In the present study, the test compound SPIII-5F inhibited the HCV RNA synthesis in Huh 5-2 Cells (SI=7) and 45% maximum protection against the replication against the replication of acutely infected SARS-CoV in Vero cells (Table 1 and 2). Isatin lead molecule (5CI-IS-AC) did not inhibit the HCV RNA synthesis and replication of SARS-CoV. Presence of sulphonamide side chain in the 3^rd^ position was essential for antiviral activity (SPIII-5H, 5Br and 5F). Further modification in the series may help in optimizing antiHCV activity.
